# Unraveling Interfacial Photoinduced Charge Transfer and Localization in CsPbBr_3_ Nanocrystals/Naphthalenediimide

**DOI:** 10.1021/acsomega.4c01651

**Published:** 2024-05-09

**Authors:** Eliane A. Morais, Maykon A. Lemes, Natalilian R. S. Souza, Amando Siuiti Ito, Evandro L. Duarte, Ronaldo S. Silva, Sergio Brochsztain, Jose A. Souza

**Affiliations:** † Center for Human and Natural Sciences, Federal University of ABC, Santo André 09210-580, São Paulo, Brazil; ‡ Engineering, Modeling and Applied Social Sciences Center, Federal University of ABC, Santo André 09280-560, Brazil; § Institute of Physics, University of São Paulo, São Paulo 05508-060, Brazil; ∥ Federal University of Sergipe, São Cristóvão 49100-000, SE, Brazil

## Abstract

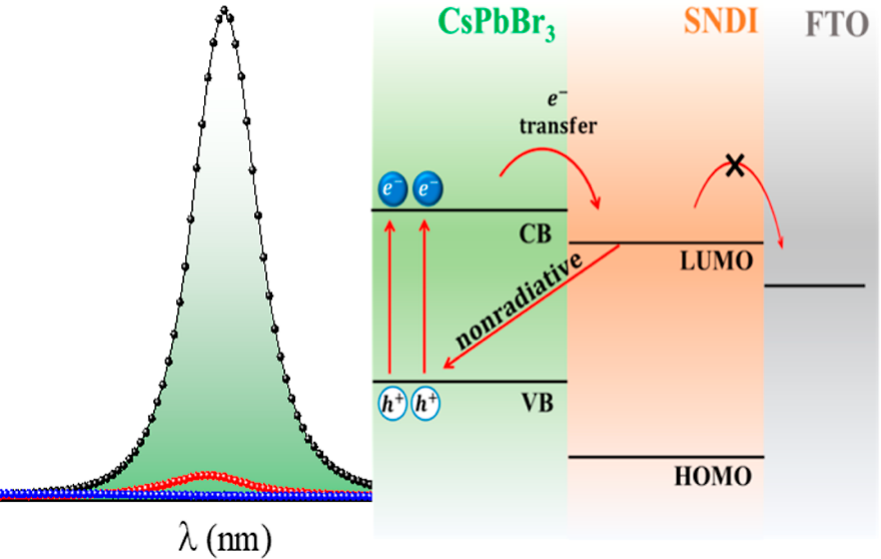

Halide perovskites have attracted much attention for energy conversion. However, efficient charge carrier generation, separation, and mobility remain the most important issues limiting the higher efficiency of solar cells. An efficient interfacial charge transfer process associated with exciton dynamics between all-inorganic CsPbBr_3_ nanocrystals and organic electron acceptors has been suggested. We observed a strong PL quenching of 78% in thin films when silane-functionalized naphthalenediimides (SNDI), used as electron-acceptors, are anchored on CsPbBr_3_ nanocrystals. Optical and structural characterizations confirm the charge transfer process without QDs degradation. The issue of whether these transferred charges are indeed available for utilization in solar cells remains uncertain. Our results reveal that the CsPbBr_3_ nanocrystals capped with these electron-acceptor SNDI molecules show a drastic increase in the electrical resistance and the absence of a photoconductivity effect. The results suggest charge transfer followed by strong localization of the charge carriers, preventing their extraction toward the electrodes of solar cell devices. We hope that this crucial aspect to attract attention and unveil a potential mechanism for charge delocalization, which could, in turn, lead to a groundbreaking enhancement in solar cell efficiency.

## Introduction

Semiconducting materials are of great interest for technological applications due to their optoelectronic properties involving the creation of exciton through light absorption and its dissociation, followed by separation into free charge carriers.^1^ Controlling the electrical transport properties involving the creation, transfer, and mobility of charge carriers can be applied in several areas, going from energy generation to catalysis. However, poor visible light harvesting, a short carrier lifetime, and mainly high carrier exciton recombination effects can hinder its practical applications.^2^ An important strategy to overcome these drawbacks is the combination of different semiconducting nanomaterials, which can be tailored to induce energy or electron transfer processes. Such hybrid-engineered interface materials can join the advantageous physical and chemical properties of each constituent, and they can convert light absorption into electrical or chemical energy. Besides tailoring the band gap energy,^3^ the key to improving efficiency in solar cells and photocatalytic activity is to suppress charge recombination and improve separation and mobility. For example, a well-defined interface in heterojunctions effectively facilitates charge transfer and suppresses the recombination of photogenerated electrons and holes, leading to higher activity and stability.^4^

Halide perovskite semiconducting materials have shown improved optical and electronic properties such as a wide absorption spectrum, tunable band gap energy, low charge recombination rates, and high charge carrier mobility, which are essential for a diversity of applications. In particular, all-inorganic cesium lead halide perovskite (CsPbX_3_, X = Cl, Br, and I) nanocrystals, with high photoluminescence (PL) quantum yields and stability, have received great attention in perovskite-based photovoltaic systems.^5−7^ As mentioned above, the conversion efficiency of their photovoltaic devices is related to the charge-transfer (CT) rate between light harvesting and hole-/electron-transporting materials. Electron-donating, -accepting, and charge-transporting sandwiched materials play an important role in the extraction of charge carriers from the active layer and decrease exciton recombination.^8^ To achieve a higher photovoltaic conversion efficiency, besides understanding the CT process across the interface, an ideal matching of interfacial engineering involving energy-level alignment is necessary. This positive synergy between materials improving photophysical properties is not easily commonly found.^9^ Nevertheless, the interfacial CT process and exciton dynamics between all-inorganic CsPbX_3_ NCs and molecular acceptors have not been fully investigated.^10^ Indeed, all-inorganic CsPbX_3_ NCs can be very important in optoelectronic devices and photocatalytic applications if an ideal counterpart is found and their interfacial charge-transfer dynamics are fully understood.^11^

The growth of a heterojunction where there is charge transfer from one part to another is a relevant matter from both technological and fundamental bases by itself due to the exciting physics and chemistry at the interface. For this reason, we have taken advantage of the electronic synergy between all-inorganic semiconductors and molecular acceptors to investigate the optoelectronic properties of QDs and a novel naphthalenediimide-based molecule in an effort to pursue highly efficient charge carrier separation materials. These naphthalenediimides (NDIs) are *n*-type organic semiconductors with great importance in electronic devices such as solar cells due to their electron affinity.^12−15^ They are rated among the best nonfullerene materials as electron-transport layers in solar cells.^16^ Moreover, NDIs can be easily reduced to stable organic radicals^17^ due to their electron-deficient nature in the aromatic nucleus, allowing the creation of a variety of hybrid materials to be used in organic electronics.^18^ Here, we report the synthesis of colloidal perovskite quantum dots decorated with an NDI derivative functionalized with silane groups, *N*,*N*′-bis(3-triethoxysilylpropyl)-1,4,5,8 naphthalenediimides (SNDI), to create local heterojunctions. SNDI features hydrolyzable silane groups, resulting in strong binding to the surface of CsPbX_3_ nanocrystals. As mentioned, it has been demonstrated that attaching electron-acceptor molecules to the surface of perovskites may facilitate charge transfer, ultimately enhancing the separation of charges into free carriers.^19^ However, the question of whether these transferred charges, driven by these molecules, are indeed available for utilization in either catalysis or solar cells remains uncertain. Here, we shed light on this issue. We have combined these electron-acceptor molecules with CsPbBr_3_ nanocrystals in the form of colloidal solutions and thin films and observed a strong PL quenching, suggesting a charge transfer process. However, DC and AC electrical transport measurements reveal a drastic increase in electrical resistance and the absence of a photoconductivity effect when the molecules are anchored. The results suggest charge transfer followed by a strong localization of the charge carriers preventing their extraction toward the metallic electrodes.

## Results and Discussion

**Figure 1:**
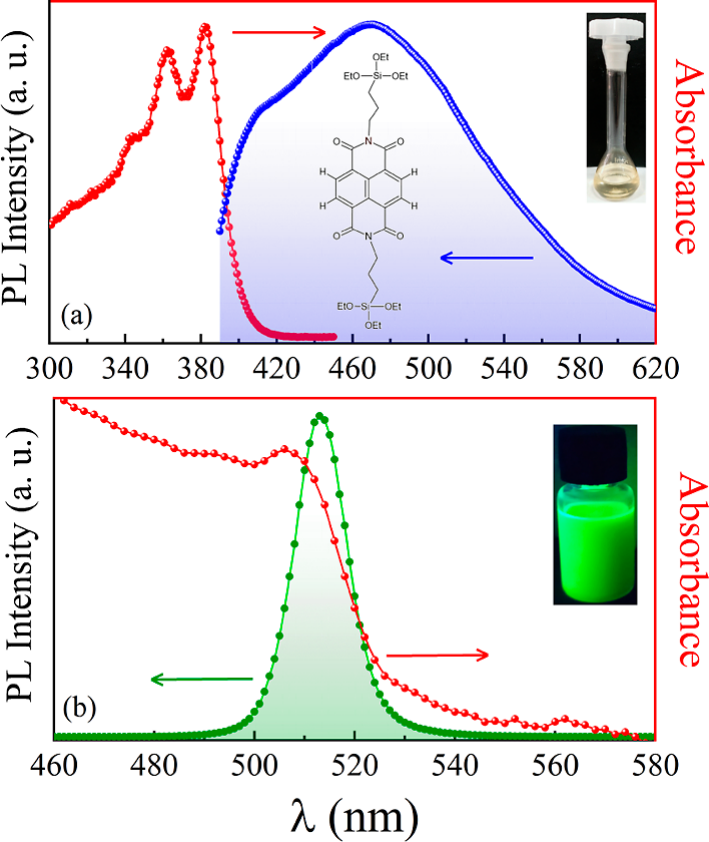
Photoluminescence and UV–visible absorption spectra of toluene solutions of (a) SNDI (λ_ex_ = 320 nm) and (b) CsPbBr_3_ QDs (λ_ex_ = 425 nm). The insets show the structure of SNDI along with its solution and the QDs colloidal solution under UV light—365 nm.

We have synthesized the colloidal quantum dot solution of CsPbBr_3_ perovskites by the hot injection method. Besides the facile technique, this synthetic method allows fast growth and nucleation of nanosized particles. The synthesis of aromatic molecules was obtained via an established procedure as mentioned above. Figure 1a,b displays the absorption and PL spectra of SNDI and CsPbBr_3_ QDs in toluene. The solution of SNDI under UV light (365 nm) is shown in the inset of Figure 1a, while a colloidal solution of CsPbBr_3_ QDs under UV (365 nm—revealing a green color) is displayed in the inset of Figure 1b. The absorption spectrum of SNDI (Figure 1a) shows the typical vibrational structure of NDI derivatives, with the most intense absorption peaking at 382 nm, a secondary maximum at 362 nm, and a shoulder at 344 nm. The structure of the SNDI is shown in the inset of Figure 1a. The PL spectrum of SNDI shows an excimer-like broad band with a maximum at 470 nm and a shoulder at 412 nm, which can be attributed to monomeric SNDI. The HOMO–LUMO energy gap of organic molecules is usually assigned at the crossing point between absorption and emission spectra, which is at 393 nm for SNDI in toluene (Figure 1a), giving E_g_ = 3.15 eV. The synthesized CsPbBr_3_ QDs exhibit a photoluminescent emission centered at 513 nm (*E* = 2.41 eV) with a fwhm of ∼13 nm. The UV–visible spectroscopy for the same sample exhibits a maximum absorption band at 508 nm (*E* = 2.44 eV).

**Figure 2:**
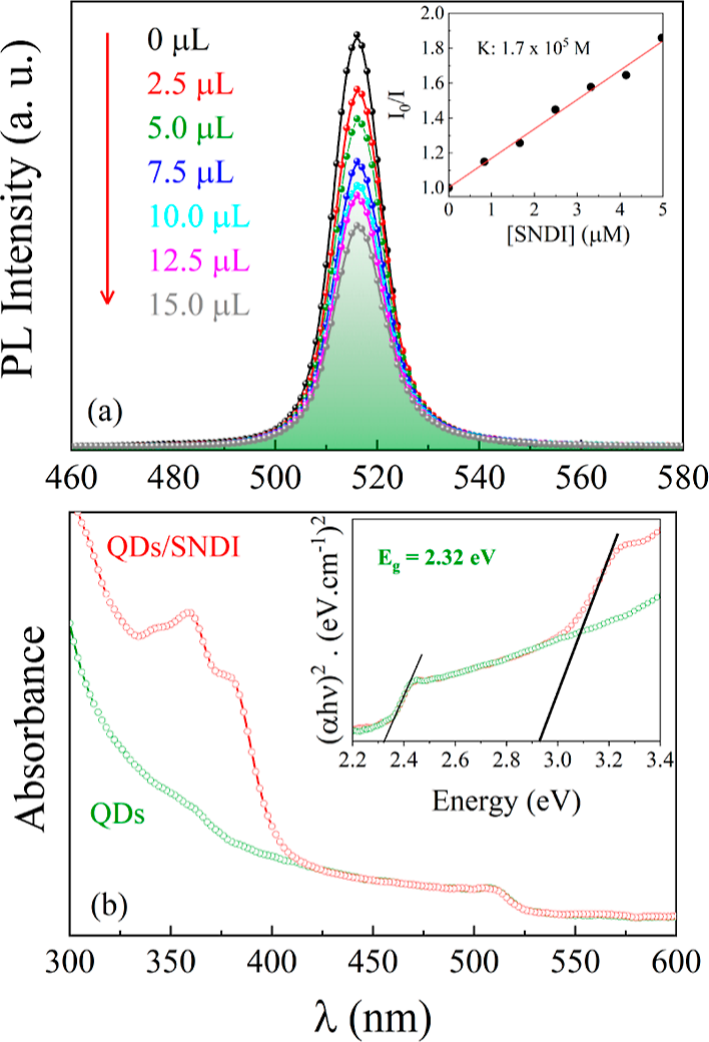
(a) PL emission measurements of CsPbBr_3_ QDs with the addition of SNDI up to 15 μL. The inset indicates a linear relationship of *I*_0_/*I* vs [SNDI] (excitation of 425 nm). (b) Absorption spectrum of the QDs of CsPbBr_3_ with the addition of 15 μL of SNDI. The inset shows a Tauc plot estimating the band gap energy of both systems. The concentrations for each measurement are SNDI (mol/L): 8.33 × 10^–7^ (2.5 μL); 1.66 × 10^–6^ (5.0 μL); 2.49 × 10^–6^ (7.5 μL); 3.32 × 10^–6^ (10.0 μL); 4.15 × 10^–6^ (12.5 μL); and 4.98 × 10^–6^ (15.0 μL).

As mentioned earlier, 1,4,5,8 naphthalenediimide (NDI) aromatic molecules are an important class of electron-accepting organic semiconductors. In order to study the synergy of SNDI and CsPbBr_3_ QDs, we have prepared solutions by titrating the QDs (2.5 μL) solution with SNDI (2.5 μL) in toluene (3 mL) placed in a quartz cuvette. Here, UV–vis absorption measurements were used to calculate the concentration of quantum dots in the solution, which was performed using Beer’s law described by the equation *A* = ε*bc*, where *A* is the absorbance (dimensionless), *b* is the optical path (cm), *c* is the concentration (mol L^–1^), and ε is the molar absorptivity (mol^–1^·L·cm^–1^). The latter is characteristic of each substance in each λ; the concentration formula used to substitute in the previous equation was *c* = (*c*_*0*_·*V*)/*V*_0_.^20^ For this calculation, we used ε = 2.42 × 10^–2^ × d^*3*^, which is the absorptivity value of the QDs of CsPbBr_3_ in toluene at a wavelength of 400 nm.^21^ Thus, the calculated concentration of the QDs in the solution was found to be 20.2 nM. After that, we performed photoluminescence analysis (Figure 2), where aliquots of 2.5 μL from the SNDI solution were added, and the PL spectrum was collected after each addition until a total of 15 μL of SNDI was reached.

As can be seen in Figure 2a, when 2.5 μL of SNDI was added to the colloidal solution of the QDs, quenching of the emission was observed. Nevertheless, a significant quenching of 54% is observed when 15 μL of SNDI is added. In the PL emission measurements, we used the excitation wavelength of 425 nm to avoid the absorption of SNDI. Such an observation suggests the presence of NDI molecules anchored on the surface of CsPbBr_3_ QDs. A quantitative analysis of PL quenching was done using the Stern–Volmer equation, *I*_0_/*I* = 1 + *K*_SV_.[SNDI], where *I*_0_ and *I* are the PL intensities before and after the addition of NDI, respectively, K_SV_ is the SV quenching constant, and [SNDI] is the concentration of the SNDI quencher. The linear behavior shown in the inset of Figure 2a produces a slope of 1.7 × 10^5^ M^–1^. This *K*_SV_ value indicates a strong interaction of SNDI with the surface of the QDs. The presence of such an interaction is anticipated to lead to photoinduced charge separation, as already observed with other electronic acceptors.^10,22,23^

As the quenching of emission in the PL of CsPbBr_3_ QDs is significant, we perform UV–vis measurements to evaluate the possible degradation of the QDs. Figure 2b shows absorption measurements of both pure CsPbBr_3_ quantum dots and CsPbBr_3_ QDs with 15.0 μL of SNDI (54% quenching of PL). The purification of the QDs solution may affect the interaction with the SNDI molecules. Usually, the purification is done by centrifugation, the solution—supernatant is discarded, and the nanoparticles are redispersed in toluene. As can be seen in Figure 2b, both samples present a narrow absorption band at 508 nm, indicating the absence of QDs degradation. The extent of emission from CsPbBr_3_ QDs at low SNDI concentrations indicates that the interaction with the quantum dots is static in nature, as observed with the Ferrocene redox couple^24^ and CdSe semiconductor colloidal solutions that interact with electron acceptors and induce photocatalytic reduction.^25,26^ According to these studies, surface electron transfer can provide insights into the photovoltaic role of semiconductor nanocrystals in solar fuel generation. As there is no efficient spectral overlap between the SNDI absorption spectrum and the CsPbBr_3_ QDs emission spectrum, we will disregard the possibility of the energy transfer process.^27^ In order to estimate the band gap energies (*E*_*g*_) of the samples, we use the Tauc plot method.^28^ The results are displayed in the inset of Figure 2b. The band gap energy of CsPbBr_3_ NCs was estimated to be *E*_*g*_ = 2.32 eV, which is consistent with the previously reported values,^29^ and for SNDI, we observe a value close to the expected around ∼3 eV.

**Figure 3:**
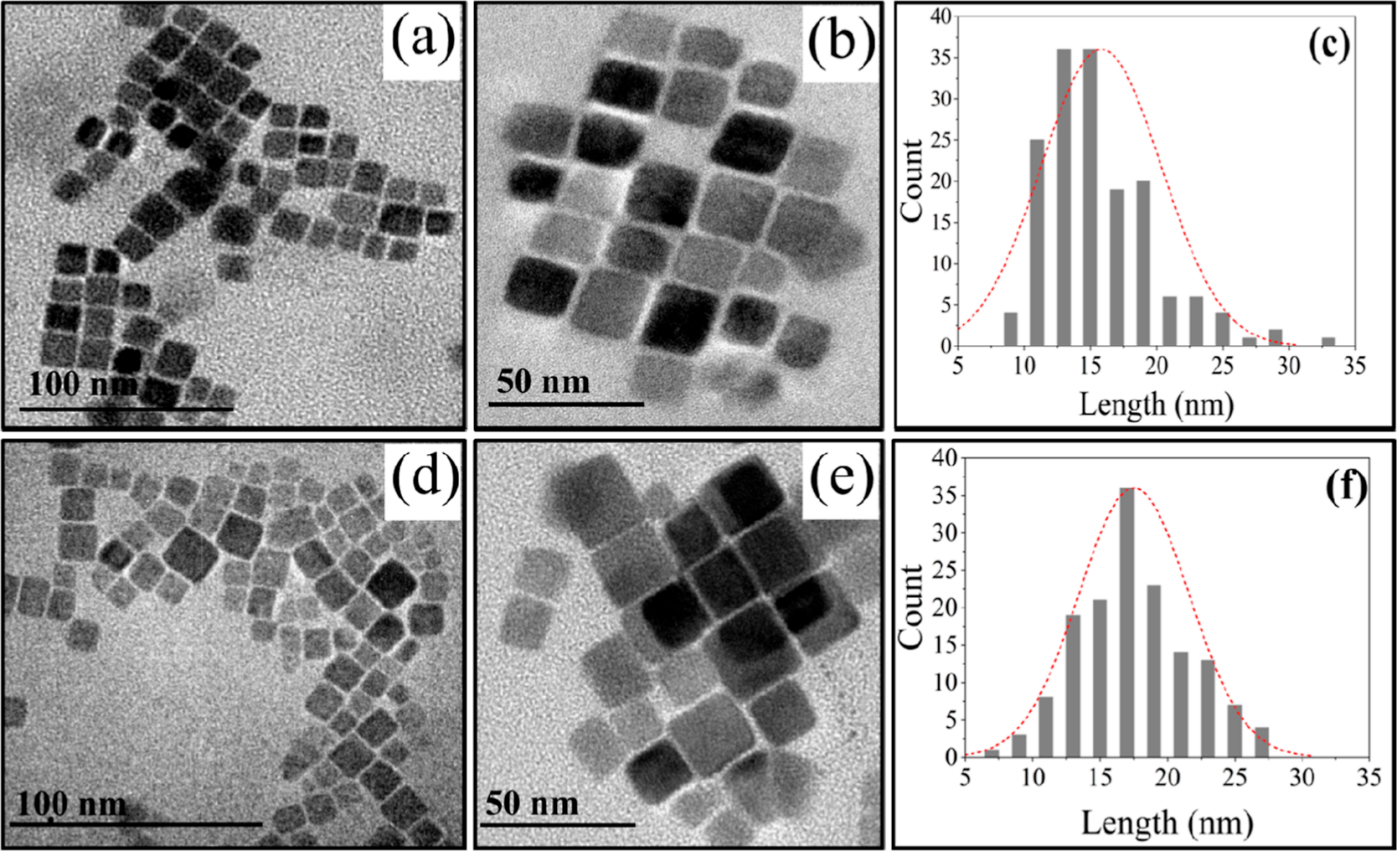
TEM images showing the morphology and size of the CsPbBr_3_ QDs (a,b) and CsPbBr_3_/SNDI (d,e). The graphics (c,f) show the particle size distribution for both samples.

Figure 3a–d displays TEM images showing the same morphology for CsPbBr_3_ QDs and CsPbBr_3_/SNDI QDs. The particles can be depicted as cuboid-like shape with a mean size distribution (the edge dimension) of 15(2) nm for the QDs and 17(2) nm for the QDs/SNDI. Notable is that the synthesized CsPbBr_3_ nanocrystals are larger than their Bohr exciton radii, exhibiting a weak quantum confinement effect.^30^ In short, from the optical analysis and TEM results, the combination of SNDI and CsPbBr_3_ QDs brings about a quenching in the photoluminescence emission, while the quencher molecules adsorbed on the surface led to a slight increase in the average size of the QDs. We have also probed the interaction of the SNDI with the QDs surface through FTIR spectroscopy (Figure S4). The characteristic bands of the oleic acid (OA) (908 and 1641 cm^–1^) and OAm (719, 991, and 1463 cm^–1^) ligands are barely seen in the SNDI/QD sample. Instead, most of the bands due to the SNDI molecule are present in SNDI/QD (marked with black stars in Figure S4), suggesting that the original ligands were replaced by SNDI. Furthermore, the band due to Si–O–C in SNDI (1058 cm^–1^) was replaced by the Si–O–Si (1118 cm^–1^) in SNDI/QD (pink stars in Figure S4), indicating hydrolysis and condensation of the triethoxysilane groups in the presence of the QDs.^31^

**Figure 4:**
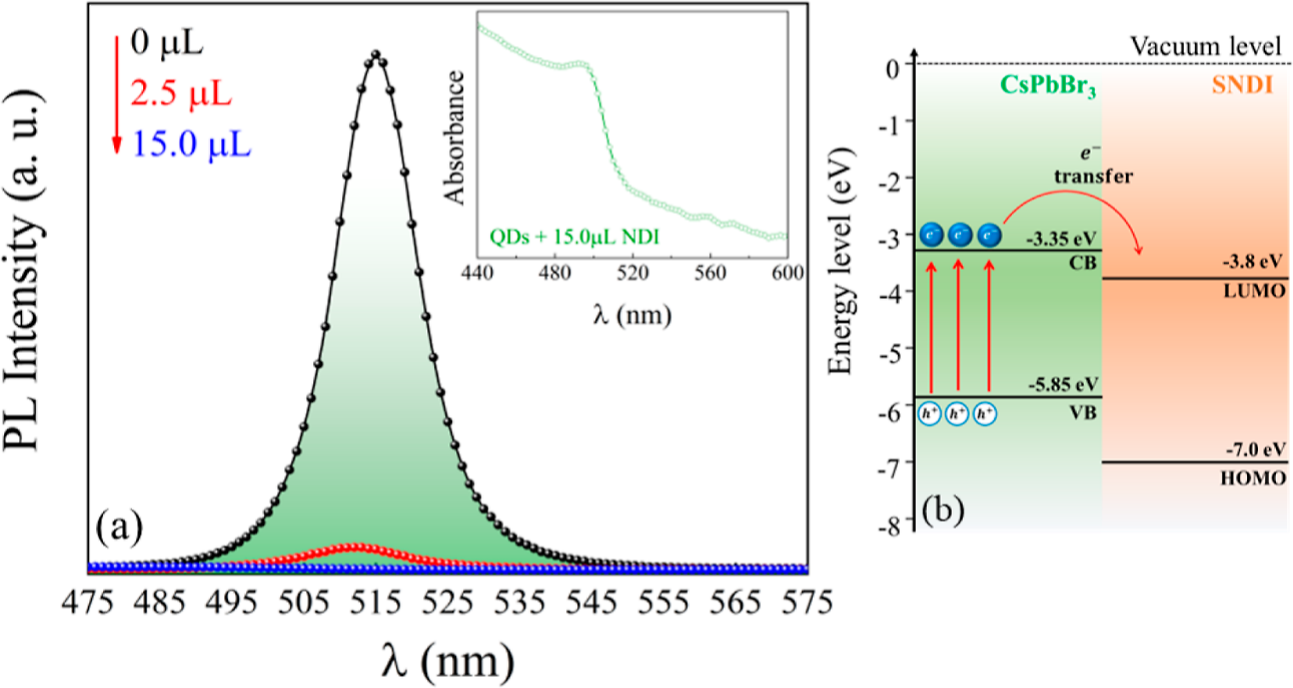
(a) Photoluminescence spectra collected in a thin film for the CsPbBr_3_ QDs pure (black), with 2.5 μL (red) and 15.0 μL (blue) of SNDI (excitation = 425 nm). The inset shows UV–vis measurements showing the absorption band at 508 nm of the QDs/SNDI thin film and (b) energy-level scheme summarizing the band structure of CsPbBr_3_ and the electronic structure of SNDI upon irradiation. The concentrations for each measurement are SNDI (mol/L): 8.33 × 10^–7^ (2.5 μL) and 4.98 × 10^–6^ (15.0 μL).

Since the potential applications of this observed charge transfer phenomenon are most likely in solid-state devices, we performed a study of the quenching of PL emission in thin films. We also discuss this effect in more detail, including time-resolved PL and charge transport through electrical resistivity characterizations. To carry out these experiments, we prepared three different solutions as mentioned in the Experimental Section. The PL measurements for the three films (pure CsPbBr_3_ QDs, with 2.5 μL, and with 15.0 μL of SNDI) are shown in Figure 4. A very strong quenching of PL is observed, suggesting complete exciton dissociation. For example, PL for the film with 2.5 μL of SNDI decreases by 78%. The inset of Figure 4 shows a pronounced absorption band at 508 nm for the hybrid sample, indicating that the QDs in the thin film with 15.0 μL SNDI are chemically stable, thus confirming that even with the complete quenching of the exciton recombination, the quantum dots remain stable. A schematic diagram showing electron affinity and ionization energy,^13^ considering the charge carrier transfer at the interface of CsPbBr_3_ and SNDI, is displayed in Figure 4b. To further verify the possible degradation of the quantum dots in the solid state after the formation of the film, we have measured X-ray diffraction, as shown in Figure S1 of the Supporting Information. We have observed that all Bragg reflections belong to the expected orthorhombic crystal phase, indicating stabilization of the CsPbBr_3_ QDs in the form of thin films and also the QDs with organic molecules, CsPbBr3 QDs/SNDI film.

When we compare the PL emission of colloidal solution and solid state as SNDI is added, a much more drastic decay is observed for the samples as a thin film. Accordingly, the distance between the semiconductor and the molecular acceptors is crucial for both electron and energy transfer. Our results are closely related to the fact that the motion degree of freedom of SNDI molecules is lower in a thin film than in a colloidal solution. Therefore, this physical state allows a massive amount of acceptor molecules anchored on the QDs surfaces and more effective contact with the CsPbBr_3_, thereby ensuring a better charge/electron transfer. The above-mentioned process suggests that the SNDI acceptors are anchored to the surface of the semiconductor. It results in a lowering of the energy levels of the molecules that facilitate the energy/charge transfer.^32^ As can be seen in Figure 4b, the estimated energy levels indicate that the SNDI LUMO is −0.45 eV lower than the conductive band and the HOMO is −1.15 eV below the valence band (VB). Such results indicate significant synergy between the perovskite semiconductor and the molecular acceptors.

Time-resolved PL analysis was employed to unravel the change in exciton recombination/dissociation and charge carrier dynamics due to the interaction between QDs and NDI molecules, as shown in Figure S2 of the Supporting Information. The PL decay intensity for the samples was fitted with a three-exponential decay function assigned to (trap-assisted) (τ_1_), exciton (τ_2_), and free charge carriers (τ_3_) recombination lifetimes.^33^ The results from the fit, along with the exponential equation, are presented in SM (see Table S1), which shows the lifetimes as well as their respective contributions (*A*). The results suggest that the main contributions of the charge dynamics among the three decay curves are attributed to the charge trapping centers (traps) (*A*_1_) and exciton recombination (*A*_2_). The average decay time decreases with the addition of the SNDI from τ_m_ = 4.3 ns to τ_m_ = 1.33 ns. This difference is associated with a change in exciton dissociation and charge dynamics, i.e., dissociation leading to charge carrier separation.

**Figure 5:**
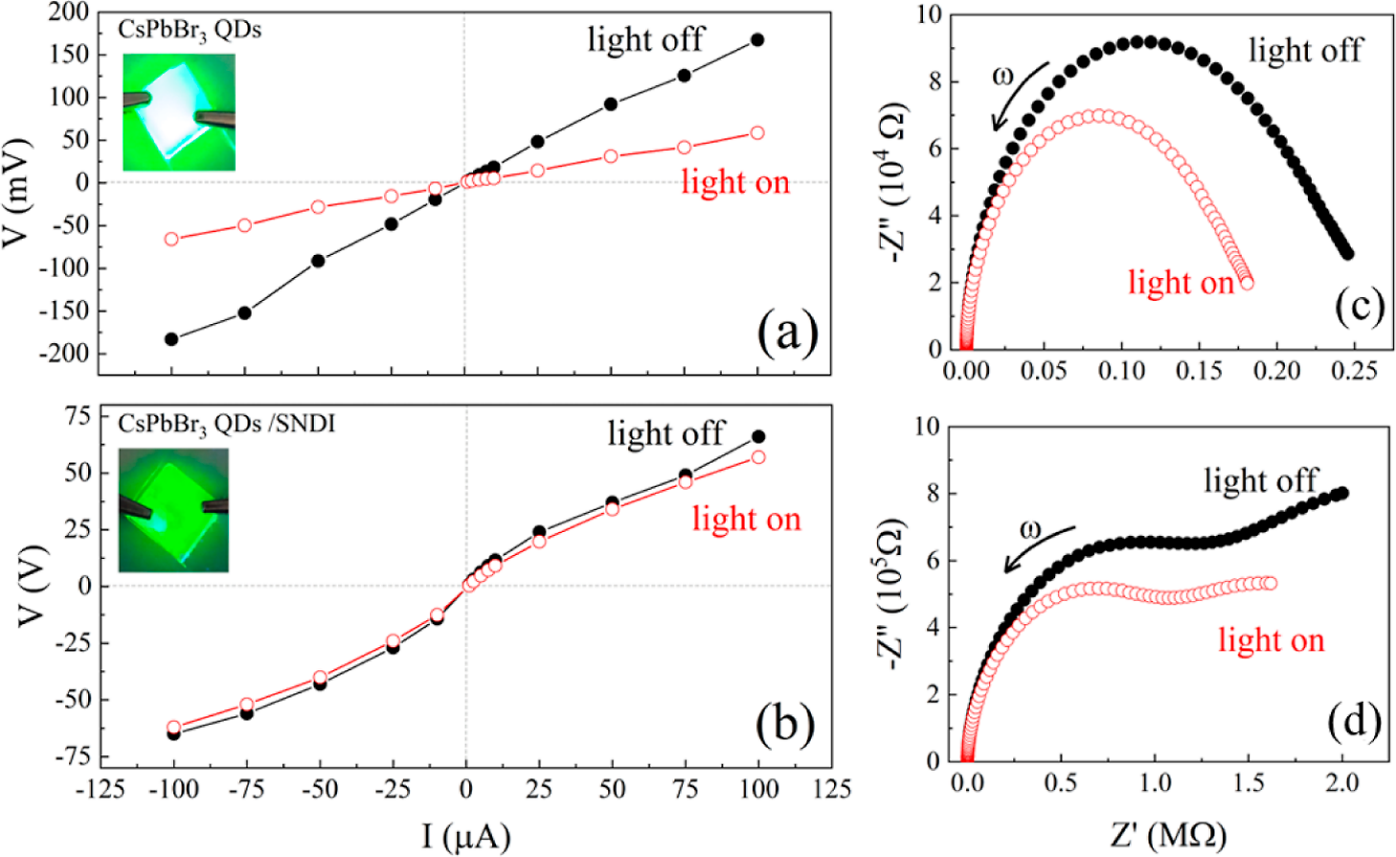
Current–voltage (*I*–*V*) curves under dark and light (1 sun from a solar simulator) for two samples: CsPbBr_3_ QDs (a) and CsPbBr_3_ QDs/SNDI (b). The inset shows a photograph of devices under UV light at 365 nm. Energy-level scheme and charge separation for the Nyquist impedance spectroscopy for the samples under conditions of dark and light illumination of 1 sun from the solar simulator for (c) CsPbBr_3_ QDs and (d) CsPbBr_3_/SNDI.

To further understand the charge transfer and photo carrier-induced dynamics in the thin films of the QDs and QDs/SNDI, electrical transport characterization was performed using direct current (DC) and impedance spectroscopy measurements.^34^ For this experiment, we set up two devices: (1) the first 40 μL of CsPbBr_3_ QDs stock solution was drop-cast five times into the FTO substrate. For each layer, the FTO substrate with the thin film of the sample was annealed at 90 °C for solvent evaporation; (2) for the second device, it was performed using the same drop casting method, however using 40 μL of the CsPbBr_3_ QDs suspension mixed with SNDI molecules. On top of the prepared thin film, another metallic glass FTO was placed, thus forming a capacitor with the sample as a dielectric sandwiched between them. The insets of Figure 5a,b show images of both devices, revealing a very bright color for the concentrated and pure CsPbBr_3_ QDs and a more shadowed film for a mixture of CsPbBr_3_ QDs/SNDI due to emission quenching. A four-probe method was used to measure the electrical resistance, which is obtained by using Ohm’s law (*V* = *R*·*I*), where *I* is a constant electrical current, *V* is the voltage drop between both substrates, and *R* is the electrical resistance of the sample measured out of the plane of the substrate. The geometric factor of all fabricated devices is very similar. Figure 5a,b shows the current–voltage (*I*–*V*) measurements for both samples in the dark and in the presence of light (1 sun) by using a solar simulator. The plot displays a very linear dependence, Figure 5a, in the *I*–*V* curves for the QDs device. The slope indicates the electrical resistance of the sample, which was found to be *R* = 1.9 kΩ. A significant drop in the slope (*R* = 0.6 kΩ) is also observed when the same sample is irradiated due to the presence of photoinduced charge carriers, showing that the charge carriers are extracted and collected toward the electrodes. For the hybrid sample, QDs with SNDI molecules, we observed a significant increase in the electrical resistivity to 0.9 MΩ, as shown in Figure 5b. These results suggest that the charge carriers are not being collected at the metallic electrodes after exciton dissociation. Furthermore, the electrical resistivity in the presence of light is slightly lower ∼0.8 MΩ, i.e., the photoelectric effect is almost absent. These results are very important and reveal that, even though we have observed a local charge transfer process, the photoinduced charge carriers are strongly localized when SNDI molecules are present. These results suggest that photoinduced electrons involve charge carrier transfer from CsPbBr_3_ to metallic glass (FTO) and their absence when SNDI is present.

The impedance spectroscopy measurements were also measured simultaneously with DC resistivity to evaluate the capacitance and relaxation dynamics of charge carriers. Figure 5c,d shows the Nyquist plots for CsPbBr_3_ QDs and CsPbBr_3_/SNDI in dark and under light illumination. The Nyquist plots for CsPbBr_3_ QDs reveal the presence of a single semicircle with a characteristic frequency of ω_0_ = 538 Hz, which is attributed predominantly to relaxation arising from the electronic transport of the particles. The imaginary part as a function of frequency is shown in Figure S3 of the SM for both samples. This characteristic frequency increases to ω_0_ = 673 Hz when the light is turned on, i.e., the charge transport is faster. The DC electrical resistance can also be found at the low-frequency limit ω ≈ 0, where the curves intercept the real axis (*Z*′). We observe a drastic variation in both imaginary and real contributions when the pure QDs are illuminated due to the photogenerated charges in the system from exciton dissociation. On the other hand, one can observe the starting of a second semicircle at low frequencies when SNDI molecules are present. It can be associated with high-resistance interparticle contribution due to the presence of molecules on the surface of the nanoparticles. This result can suggest that the presence of the molecules decorating the QDs increases the grain boundary resistance contribution. Intergrain contributions are observed to be very important in the transport properties of these halide perovskites.^35^ The electrical resistance for these hybrid samples is higher, as observed in DC results. A small variation is also observed for CsPbBr_3_/SNDI when comparing dark and under light irradiation. The characteristic frequency changes from ω_0_ = 300 to 378 Hz when the light is turned on. All of the parameters from impedance spectroscopy measurements are displayed in Table S2 of Supporting Information. A more detailed study involving the capacitance and dielectric constant of these two devices will be published elsewhere.

**Figure 6:**
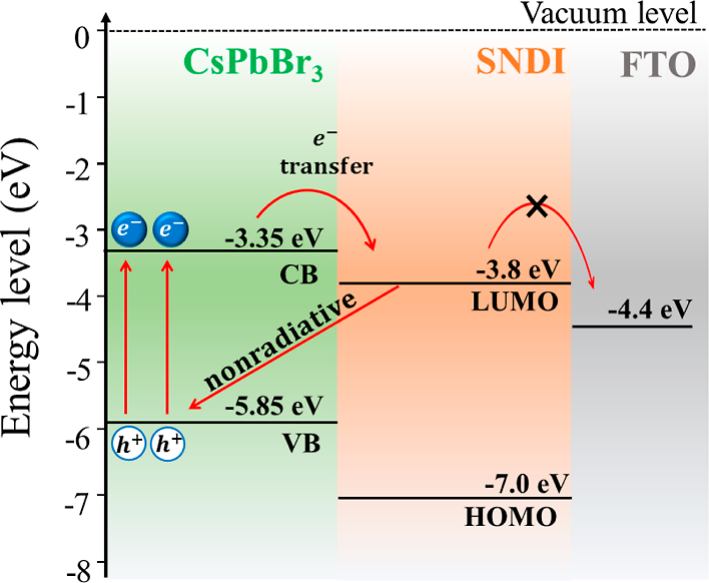
Schematic illustration of the energy-level diagram of QDs, SNDI, and FTO, along with transfer and recombination pathways upon irradiation.

To achieve high charge separation and mobility, a detailed understanding of the charge transfer process across the interface and favorable interfacial energy-level alignment is necessary. The interfacial dynamic process and exciton dynamics between all-inorganic CsPbX_3_ NCs and molecular acceptors have not been fully understood so far. The sequential achievement of light absorption by the perovskites, exciton formation and dissociation, charge extraction, and collection of free carriers at the metallic electrodes ultimately decides the successful performance of a photovoltaic device. Figure 6 shows a schematic illustration of the energy-level diagram of QDs, SNDI, and FTO, along with the transfer/recombination pathways. It is suggested that when the hybrid sample (CsPbBr_3_/SNDI) is irradiated with 425 nm, the electrons from the VB are promoted to the conduction band of the QDs, afterward, to the LUMO of SNDI molecules. In the sequence, even though there is a favorable interfacial energy-level alignment with FTO, the photoinduced charges are not transferred to the FTO electrode. Therefore, our results indicate that this state is strongly localized, and the photoinduced electrons cannot be collected at the FTO metallic substrate. Eventually, the electrons are transferred back to the VB of the quantum dots in nonradiative recombination, as illustrated in Figure 6.

## Conclusions

Electron-accepting molecules used as charge-separating materials can play an important role in the extraction of charge carriers, opening the possibility to increase solar cell efficiency. In our study, we have successfully synthesized both CsPbBr_3_ QDs and SNDI molecules. The interaction of SNDI with QDs proved to be highly effective in quenching the luminescence in both colloidal solutions and thin films. Notably, in the latter, the PL quenching was particularly more pronounced, with a reduction in intensity of 78% without degradation of the QDs. The CsPbBr_3_ QDs and CsPbBr_3_ QDs/SNDI samples exhibited a uniform size distribution, 15(2) nm for the former and 17(2) nm for the latter, featuring a cubic-shaped particle morphology. The time-resolved PL analyses showed that the major contributions of charge dynamics among the three decays are attributed to the charge trapping centers and exciton recombinations. The DC and AC transport measurements showed a significant increase in the electrical resistivity in hybrid samples (comprising CsPbBr_3_ quantum dots and SNDI). Additionally, no clear evidence of enhanced photoconductivity effects was observed, implying a robust localization of the photoinduced charge carriers. Upon irradiation, the electrons in the conduction band of the QDs are transferred to the LUMO energy level of SNDI molecules, but despite the favorable alignment of interfacial energy levels, our findings indicate that the photoinduced charges do not undergo transfer to the FTO metallic electrodes. Consequently, our results suggest that these transferred charges are strongly localized and unable to be collected by the FTO metallic substrate. We have proposed a mechanism to elucidate the charge transfer process and the factors contributing to their localization, which hinder their extraction toward the electrodes. We expect that this vital aspect will draw attention and reveal potential pathways for charge delocalization, ultimately paving the way for a groundbreaking improvement in solar cell efficiency.

## Materials and Methods

### Chemicals

All chemicals were purchased from Sigma-Aldrich and Synth and were used without further purification. Cesium carbonate (Cs_2_CO_3_, Sigma-Aldrich, 99.9%), OA (Sigma-Aldrich, 90%), 1-octatdecene (ODE, Sigma-Aldrich, 90%), oleylamine (OAm, Sigma-Aldrich, 70%), lead bromide (PbBr_2_, Alfa Aesar, 99.99%), and toluene (Sigma-Aldrich, 99.8%). SNDI was synthesized through the reaction between 1,4,5,8-naphthalenetetracarboxylic dianhydride and 3-aminopropyltriethoxysilane according to the reported procedure.^36,37^

### Preparation of Cs-Oleate

To prepare lead halide perovskite QDs via the hot injection method, a previous step, including preparation of the Cs source, was performed. Thus, Cs-oleate was prepared by transferring Cs_2_CO_3_ (0.407 g), OA (1.25 mL), and ODE (20 mL) into a 25 mL 3-neck round-bottom flask under vacuum and at a temperature of 120 °C for 1 h. The solution was then heated to 150 °C under an argon atmosphere until all Cs_2_CO_3_ reacted with OA. Because the Cs-oleate precipitates out of the ODE at room temperature, the solution must be preheated to 90 °C before use in the synthesis of QDs.

### Synthesis of CsPbBr_3_ QDs

This synthesis was prepared via the hot injection method in accordance with a literature procedure.^38−40^ 5 mL of ODE and 0.188 mmol of PbBr_2_ (0.069 g) for CsPbBr_3_ QDs were added into a 25 mL 3-neck round-bottom flask at 125 °C, under vacuum, for 1 h. After that, 0.5 mL of OA and 0.5 mL of OAm were injected at 120 °C under argon for 1 h. With the complete solubilization of PbBr_2_ salt, the temperature was then raised to 190 °C, and 0.4 mL of Cs-oleate solution was quickly injected. Immediately after 1 min, the mixture was cooled down in a water bath. After reacting all the precursors, the solution was instantly cooled down to 0° using an ice bath where the QDs were immediately formed. The resultant aggregated QDs were separated by centrifugation, and the supernatant was discarded. The precipitate was redispersed in dried toluene for all solutions.

### Preparation of SNDI Stock Solution

A 1 mM NDI stock solution was prepared by dissolving 6.7 mg of SNDI in 10 mL of toluene.

### PL Quenching of a QD Colloidal Solution with SNDI

2.5 μL of the QDs stock solution was added to 3 mL of toluene in a quartz cuvette, and the initial PL spectrum was registered. Aliquots of 2.5 μL from the SNDI stock solution were then added (up to 15 μL), and the PL was measured again after each addition.

### Preparation of QDs/SNDI Thin Films

To produce thin films, three solutions were separately prepared and dropwise added in quartz substrates; the first solution was prepared by combining 3 mL of toluene and 2.5 μL of QDs. The second one combines 3 mL of toluene, 2.5 μL of QDs, and 2.5 μL of SNDI. And for the third solution, a mix of 3 mL of toluene, 2.5 μL of QDs, and 15 μL of SNDI. The dry thin films were obtained after complete solvent evaporation.

### Experimental Characterization

Powder X-ray diffraction data were obtained using a diffractometer STADI-P model of Stoe with CuK_α1_ radiation whose wavelength is λ = 1.5406 Å CuKα, operating in the 40 kV/40 mA regime in a scanning interval of 2θ between 3 and 60° with a speed of 2°/min. To analyze the sample surface morphology and topography, transmission electron microscopy (TEM) images were obtained from a JEM1400 plus equipment from Jeol Thermo Scientific operating at 120 kV. Optical analysis was performed through UV–visible spectroscopy using an Evolution 220 Thermo Fisher spectrophotometer and photoluminescence using a static PL-spectrophotometer (Horiba fluorescence). For the time-resolved fluorescence measurements, the excitation source used was a titanium-sapphire laser Tsunami 3950 Spectra Physics, pumped by a solid-state laser Millenia Pro model 10sJS Spectra Physics. The repetition rate was set to 8.0 MHz using a pulse picker (Spectra Physics, model 3980-25). The Tsunami was set to give an output of 840 nm, and a second harmonic generator BBO crystal (GWN-23PL Spectra Physics) was used to generate the excitation beam at 422 nm. This beam was directed to a spectrofluorometer, model FL900CDT (UK). The emitted light was detected by a refrigerated microchannel plate photomultiplier (Hamamatsu R3809U) at 90° from the excitation beam. The emission wavelength of 516 nm was selected by a monochromator. The electrical transport (Keithley 2410 Sourcemeter) and impedance spectroscopy (SOLARTRON Impedance Analyze SI1260 coupled to a dielectric interface) measurements were performed by using a four-probe method carried out with an LED Solar Simulator (LSH-7320) in the 400–1100 nm wavelength range. The maximum output power is 110 mW/cm^2^, which is equivalent to 1.1 sun.

## Supporting Information Available

Additional XRD patterns, photoluminescence lifetime, values of lifetimes, the Nyquist impedance spectroscopy, and FTIR spectra for all samples (PDF)
