# Supplementary material for: Unraveling Interfacial Photoinduced Charge Transfer and Localization in CsPbBr3 Nanocrystals/Naphthalenediimide
Source: ACS Omega. 2024 May 9;9(20):22296–304. doi: 10.1021/acsomega.4c01651 (PMC11112556; doi:10.1021/acsomega.4c01651)
Supplement: Supplementary file 1 — ao4c01651_si_001.pdf [file ao4c01651_si_001.pdf]

Supplementary Material

Unraveling Interfacial Photo-induced Charge Transfer and Localization in $CsPbBr_3$ Nanocrystals/Naphthalenediimide

Eliane A. Morais[a], Maykon A. Lemes[a], Natalilian R. S. Souza[a], Amando Siuiti Ito[b,c], Evandro L. Duarte[c], Ronaldo S. Silva[d], Sergio Brochsztain[b], Jose A. Souza[a*]

[a]Center for Human and Natural Sciences, Federal University of ABC, Brazil, Santo André, 09210-580 SP, Brazil
[b]Engineering, Modeling and Applied Social Sciences Center, Federal University of ABC, Brazil.
[c]Institute of Physics, University of Sao Paulo
[d]Federal University of Sergipe, São Cristóvão, 49100-000, SE, Brazil

Corresp. Author: joseantonio.souza@ufabc.edu.br

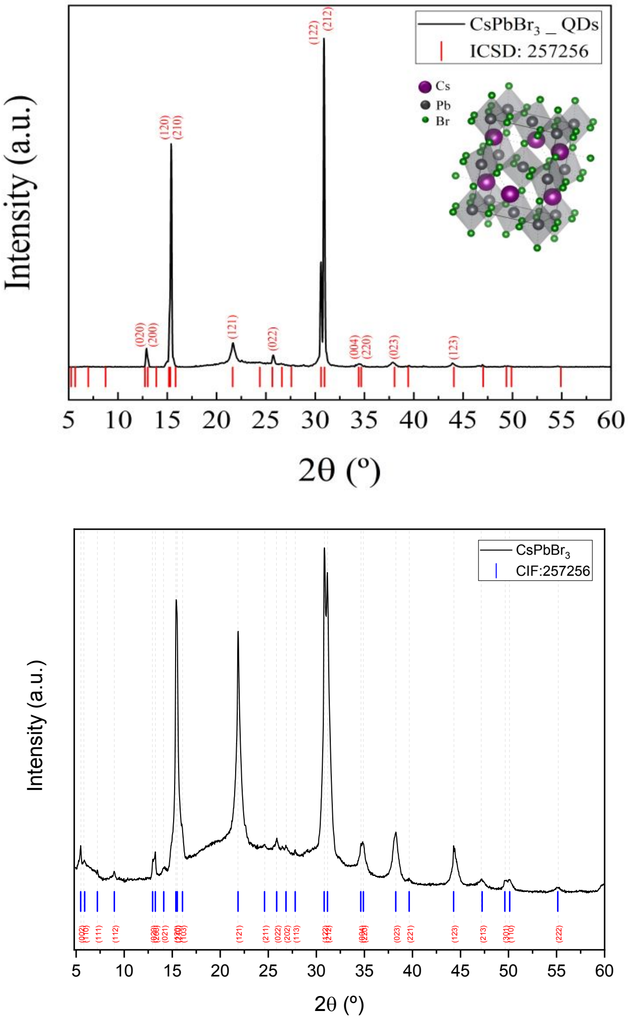

Figure S1: Upper panel X-ray powder diffraction of the $CsPbBr_3$_QDs in the form of thin film showing structural stabilization on the substrate and below the QDs with organic molecules, CsPbBr3 QDs/SNDI film.

The PL decay intensity for the samples was fitted with a three-exponential decay function assigned to (trap-assisted) (τ_1), exciton (τ_2), and free charges carriers (τ_3) recombination lifetimes using the equation:

$$I(t) = I_0 + \sum_i A_i \exp\left(-t/\tau_i\right)$$

where I (t) is the intensity of the light emitted by the material after of excitation ceased, I_0 is the intensity at time t = 0, and τ decay time.

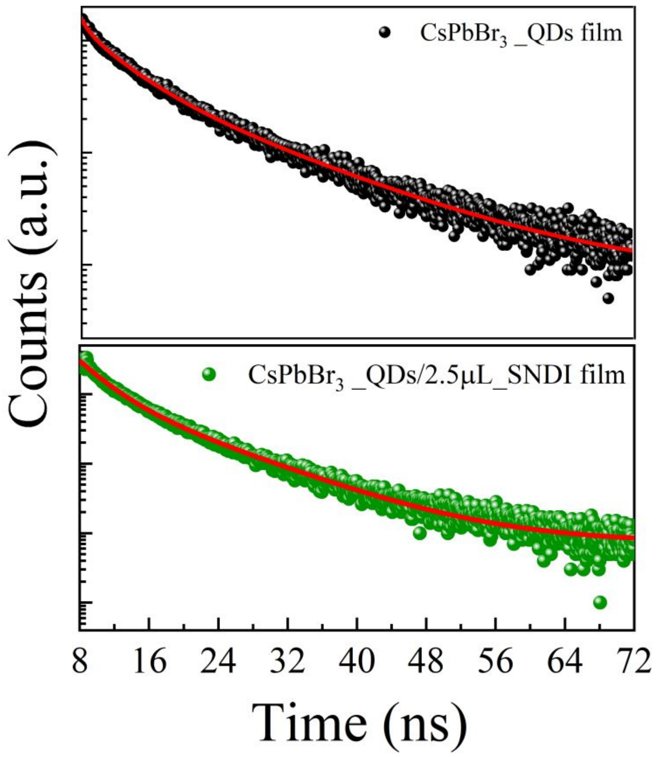

Figure S2: Photoluminescence lifetime of $CsPbBr_3$ QDs pure (black), with 2.5 μL of SNDI (green) of SNDI (excitation = 422 nm) in thin film. The red line is a three-exponential fit.

Table S1: Values of lifetimes (τ) and contributions referring to the exponential pre-factor (*A*) for the films of QDs and QDs/SNDI

**Samples**	$\tau_1\ (ns)$	$A_1\ (\%)$	$\tau_2\ (ns)$	$A_2\ (\%)$	$\tau_3\ (ns)$	$A_3\ (\%)$	$\tau_m\ (ns)$
**$CsPbBr_3$_QDs**	3.04	81.3	8.67	16.4	22.01	2.3	4.3
**$CsPbBr_3$_QDs/2.5μL _SNDI**	1.27	98.6	5.01	1.3	13.47	0.1	1.33

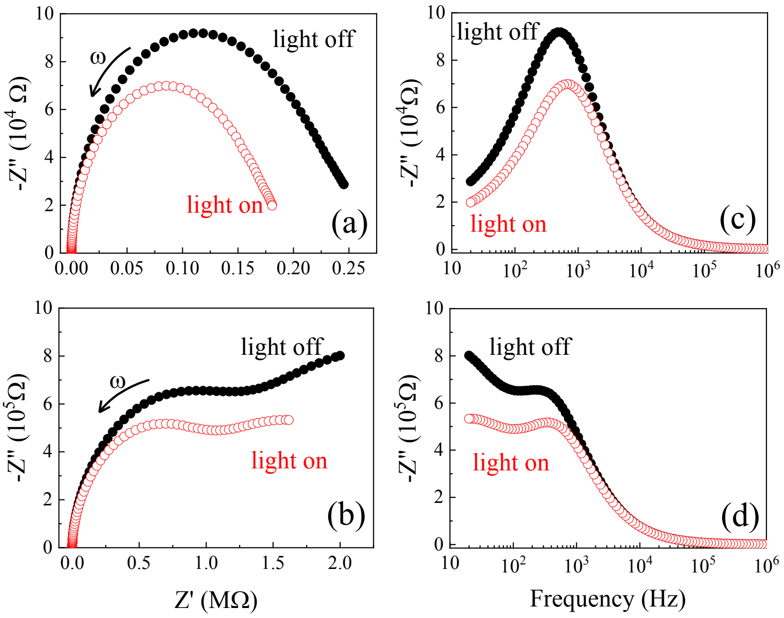

Figure S3: The Nyquist impedance spectroscopy for the samples under conditions of dark and light illumination of 1 sun from solar simulator for (a) $CsPbBr_3$ QDs and (b) $CsPbBr_3$/SNDI. Frequency dependence of the imaginary (Z") plot for (c) $CsPbBr_3$ and (d) $CsPbBr_3$/SNDI.

We have perfomed impedance spectroscopy measurements to evaluate the capacitance and relaxation dynamic of charge carriers. We have used the equivalent circuit:

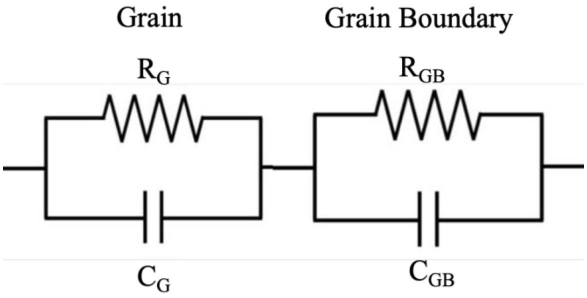

Scheme S1: Equivalent circuit for the grain and grain boundary contributions.

The semicircle at high frequency is attributed to relaxation arising from the electronic transport of the particles. The values are in the table below and also in the Supplemental Materials. The second semicircle at low frequencies when SNDI molecules are present is associated with high resistance interparticle contribution due to the presence of molecules on the surface of the nanoparticles.

Table S2. Resistance, capacitance, and relaxation frequency values

	$CsPbBr_3$ QDs		$CsPbBr_3$ QDs/SNDI	
	**Dark**	**Light**	**Dark**	**Light**
**AC**	R=2.29x10⁵ Ω	R=1.748x10⁵ Ω	R=1.546x10⁶ Ω	R=1.262x10⁶ Ω
	C=3.01x10¹⁰ F	C=2.98x10¹⁰ F	C=1.30x10⁹ F	C=1.31x10⁹ F
	ω_0=534.7x10³ Hz	ω_0=673.2x10² Hz	ω_0=424.7x10² Hz	ω_0=300.7x10² Hz
**DC**	R=1.9x10³ Ω	R=0.6x10³ Ω	R=0.7x10⁶ Ω	R=0.6x10⁶ Ω

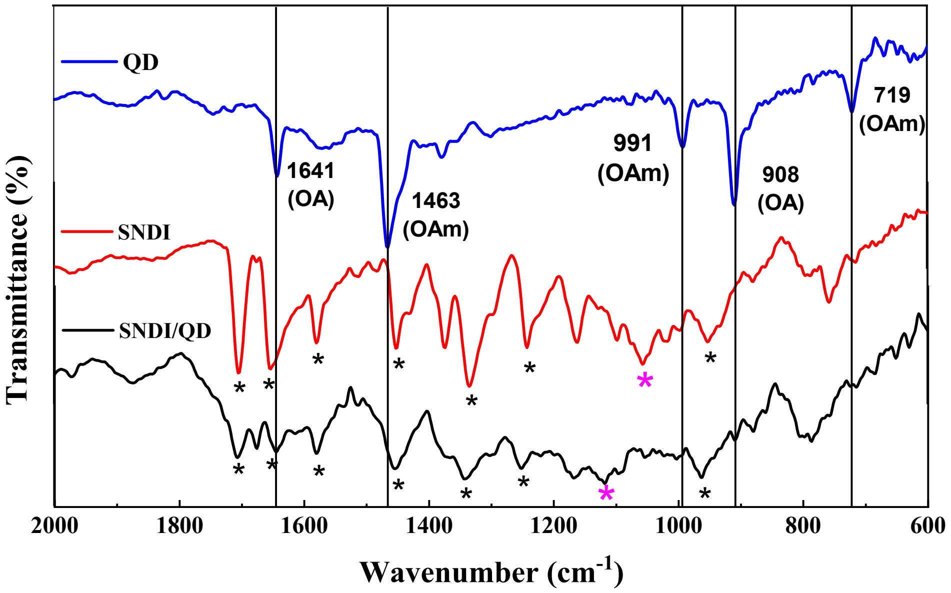

Figure S4: FTIR spectra for QDs, SNDI, and SNDI/QDs.
